# Physiological variability in CSF motion using magnetic resonance time spatial labeling inversion pulse (Time-SLIP) - real time imaging

**DOI:** 10.1186/2045-8118-12-S1-O27

**Published:** 2015-09-18

**Authors:** Shinya Yamada

**Affiliations:** 1 Toshiba Rinkan Hospital, Japan

## Introduction

The ideal tracer for studying CSF dynamics is CSF itself. In time spatial labeling inversion pulse (Time-SLIP), MR radiofrequency pulses convert specific volumes of CSF into an endogenous tracer. CSF dynamics can then be observed under physiological and pathophysiological conditions. A gate-free and fast image acquisition technique like Time-SLIP is necessary to visualize natural CSF motion, whose behavior varies with cardiac pulsation and respiration.

## Aim

To study physiological variability in CSF motion using the MRI Time-SILP method.

## Methods

A real-time Time-SLIP balanced steady state free precession (bSSFP) sequence was used on 1.5T and 3T MRI scanners. Acquisition time for each image was approximately 130msec. Serial images were obtained one to five seconds after the labeling pulse. Pulsatile CSF motions over four to five cardiac strokes were analyzed.

## Result

Considerable pulsatile CSF motion variability was observed in normal physiological brains as well as pathophysiological (hydrocephalus) brains.

## Conclusion

Real-time MR imaging is necessary to investigate natural pulsatile CSF motion. Averaging over multiple pulsatile CSF motions potentially wipes out natural physiological variability in CSF motion.
